# Influence of MgO-Lignin Dual Component Additives on Selected Properties of Low Density Polyethylene

**DOI:** 10.3390/polym12051156

**Published:** 2020-05-18

**Authors:** Karol Bula, Grzegorz Kubicki, Adam Kubiak, Teofil Jesionowski, Łukasz Klapiszewski

**Affiliations:** 1 Institute of Materials Technology, Faculty of Mechanical Engineering, Poznan University of Technology, PL-60965 Poznan, Poland; grzegorz.kubicki@student.put.poznan.pl; 2 Institute of Chemical Technology and Engineering, Faculty of Chemical Technology, Poznan University of Technology, PL-60965 Poznan, Poland; adam.l.kubiak@doctorate.put.poznan.pl (A.K.); teofil.jesionowski@put.poznan.pl (T.J.)

**Keywords:** MgO-lignin hybrid filler, low-density polyethylene, thermoforming process, microstructure properties, wettability

## Abstract

The presented study describes the application of lignin-based dual component fillers into low-density polyethylene (LDPE) and an examination of their selected properties. The main experimental procedure was focused on the preparation of thin sheet films using polyethylene and its composites with 5% by wt. of fillers: MgO, MgO-lignin dual phase systems with varying amounts of lignin and pristine lignin. Analysis of morphology revealed that elongated voids appeared in the structure for hybrid filler with a higher content of lignin (min. 50% by wt. of lignin versus MgO) and also for pristine lignin. Moreover, the prepared sheets were subjected to the thermoforming process by using the positive forming method (male mold). The thermoforming ability of all composites was evaluated by means of a comparison of wall thickness distribution on thermoformed shapes. The most noticeable percentage of wall thinning occurred for films which consisted of LDPE/MgO-lignin (5:1 wt./wt.) composite. In contrast, the best material arrangement and the highest mean percentage wall thickness were observed in the case of the shape formed with LDPE/MgO-lignin (1:5 wt./wt.). In addition, as part of research studies, the measurements of the contact angle have been conducted. The analysed LDPE films, in particular LDPE/MgO-L, have been recognized as materials with high wettability.

## 1. Introduction

As a natural polymer, lignin has currently become a component with huge potential for adaptation in practical polymeric applications. Numerous advantages of lignin include, e.g., its low cost, UV-absorption, anti-oxidant activity, and capability of polymer surface energy modification, particularly if we take into account its application in the polymer industry [1,2,3,4,5,6]. Other benefits of this bio-filler compared to synthetic fibers and inorganic particles are related to its low abrasion against plasticizing units during melt processing and lower density in comparison to mineral powders [5,6,7]. Moreover, the high content of the hydroxyl group allows for the functionalization of lignin with silanes to enhance the affinity to non-polar polymer matrices [8,9,10]. Therefore, pristine lignin and lignosulfonates are used very frequently as active fillers in different polymeric systems. Among commonly-used petrochemical polymeric matrices, polyethylene [11,12], polypropylene [13,14], and polystyrene [15] are utilized in combination with lignin. Additionally, biodegradable materials such as PLA (polylactic acid) [16,17] or PBAT (polybutylene adipate-co-terephthalate) [18] are usable matrices combined with lignin.

A common problem with natural additives derived from biomass is associated with the prevention of their thermal degradation during processing in hot plasticizing units. Some typical problems related to the processing of these materials are connected with their hydrophilic nature and with their poor thermal resistance, so processing temperatures should be kept below approximately 200 °C [19,20,21]. The limitation of temperature and the residence time is connected with the extrusion of thermoplastics with bio-based components. Usually, extruder plasticizing units are far larger than in the case of injection molding, and their length is even 48 L/D, where L/D is a factor (L-screw length, D-screw diameter) which describes the length of screw working in extruder. Moreover, extrusion of the profiles requires the use of shaping heads, known as static elements, in case of which the mean residence time could rise, and therefore, lignocellulosic materials are exposed to overheating, which may result in the occurrence of carbon black on the profile. A possible solution to this technological problem is to formulate bi-component/dual phase fillers using a bio-based substrate (such as lignin) and a second, thermally-resistant component, such as inorganic oxides. In our research team, we have synthesized a few prospective so-called dual phase fillers via mechanical alloying, in case of which lignin was connected with selected oxides: SiO_2_, Al_2_O_3_, ZnO [22,23,24]. One of the expected consequences of combining such materials is a shift of temperature decomposition above the processing window of the thermoplastic matrix. In all formulations, the thermal stability of bio-based fillers was improved as determined by means of detected mass loss with the TGA method [25].

Therefore, it seems to be very interesting to combine lignin with other inorganic compounds, including highly thermally stable MgO [26,27,28,29]. This gives the opportunity to create functional composites for advanced applications. 

Many reports dealing with lignin and polymers have been devoted solely to the investigation of structural and mechanical properties of widely-mixed lignin with thermoplastics. Despite the importance of such results, there is a lack of information regarding the validation of technological parameters of such interesting materials [30,31,32,33]. Most authors would rather avoid the discussion regarding technological problems associated with the processing of materials in which lignin and its mixtures are subjected to thermal treatment. A basic test of melt volume flow index (MFI) or the relation between lignin content and rheological properties seems to be very insufficient [34]. 

Additionally, the introduction of even a low concentration of lignin into polyethylene may contribute to prolonged UV stability due to the ability of phenolic guaiacyl and phenolic syringyl units to scavenge free radicals [35]. Such antioxidant effects of lignin in LDPE and high-density polyethylene (HDPE) systems were confirmed by Pucciariello et al. [36] and Levon et al. [37], respectively. In consequence, a low permeation of UV light and the antioxidant properties of lignin determined its application as an effective modifier for food packaging films [38,39].

Therefore, from a practical point of view, it is necessary to perform fundamental tests that can reveal the technological properties and allow us to assess the potential implementation of polymer/lignin compounds to semi-finished products. In this work, we have prepared thin sheet films using low-density polyethylene, pristine lignin, and dual MgO-lignin filler. Semi-finished sheets were subjected to thermoforming trials to reveal their processability, established based on wall thinning during thermoforming. The practical aim was to compare the wall thickness distribution of thermoformed cups based on LDPE and its composites with lignin and MgO-lignin composites. Moreover, the morphologies of tested composites were investigated using a scanning electron microscope (SEM) and an optical microscope (OM) and finally were discussed. We would like to emphasize that this report is a continuation of an already-published paper in which we have extensively described the LDPE/MgO-lignin hybrid materials in terms of their physicochemical and dispersive-microstructural properties [26].

## 2. Materials and Methods

### 2.1. Materials

Magnesium oxide (MgO)-type caustic calcined magnesia (CCM) with purity ≥99% and kraft lignin (L), average *M*_w_ ~10,000, were used in the presented research. Both reagents were supplied by Sigma Aldrich *(*Steinheim am Albuch, Germany*).* Low-density polyethylene—LDPE, (Malen E FGNX 23-D006 BasellOrlen Polyolefins Sp. zo.o. (Płock, Poland)) with a melt flow rate (MFR) of 0.8 g/10 min at 190 °C, together with polyethylene-*graft*-maleic anhydride (PE–*g*–MAH) supplied by Sigma-Aldrich (Steinheim am Albuch, Germany) with 0.5% maleic anhydride content, was used as a polymer matrix and polar agent, respectively.

### 2.2. Preparation of MgO-Lignin Hybrid Fillers

MgO-lignin hybrid materials (MgO-L) were prepared using a mechanical method. For this purpose, appropriate components (magnesium oxide and lignin in predetermined weight ratios—1:5 wt./wt. 1:1 wt./wt. and 5:1 wt./wt.) were mechanically combined by grinding and homogenizing the system in a planetary ball mill (Fritsch GmbH, Idar-Oberstein, Germany) for 2 h. Details regarding the process for obtaining inorganic-organic hybrid materials have already been included in our previous publications [22,23,24,25,26,27,40,41]. Additionally, we included detailed physicochemical and microstructural characteristics of the obtained MgO-lignin products and pristine components in our earlier reports [26,27]. In these articles, we presented the analysis of Fourier transform infrared spectroscopy for the used MgO, lignin, and obtained hybrid fillers. Furthermore, we examined the thermal stability of all products, presented scanning electron microscope microphotographs, and determined the dispersion data.

### 2.3. Polyethylene/PE-g-MAH/MgO-Lignin Formulation and Processing

The melt mixing of LDPE/PE–*g*–MAH/MgO-lignin hybrid composites was realized using a laboratory co-rotating twin-screw extruder Zamak 16/40 EHD (Zamak Mercator Sp. zo.o., Skawina, Poland), with the screw diameter of 16 mm and L/D ratio value equal to 40. The temperature range of the twin-screw extruder barrel varied from 160 to 195 °C. For the final processing step concerning the shaping of thin films, the neat LDPE and LDPE/PE-g-MAH/MgO-lignin hybrid composites were processed in a laboratory cast film line (Remi-Plast, Czerwonak, Poland). During film processing, the main chill roller was kept at a constant temperature of 45 °C by the external cooling chiller. The temperature of the extruder heating sections was set as: 160, 175, 180, 185, 190, 200 °C, and the screw rotation was set as 70 rpm. The thin sheet films were cast formed with the chill roller operated at a 2 m/min of its speed and the continuous sheet was drawn away by using the two tracer rolls coated with rubber. Additional details regarding the apparatus and processing conditions concerning directly melt compounding (twin-screw plasticizing unit design) and thin sheet film processing descriptions were available in Reference [35] and in References [26,36], respectively.

Finally, the thin sheet films of LDPE and its composites with 5% by wt. of prepared fillers (MgO, LDPE/MgO-lignin with varying amount of lignin, pristine lignin) together with 2% by wt. of PE–*g*–MAH were subjected to the testing procedure.

### 2.4. Microstructural Investigations

For the monitoring of the microstructure and assessment of the dispersibility of different hybrid fillers and pristine lignin, an optical microscope Eclipse E 400 was utilized (Nikon, Tokyo, Japan). Thin films were cut from long sheets and subjected to microscopic observation in transmittance mode. Moreover, scanning electron microscopy (SEM) was carried out for the examination of aggregate sizes and shapes using the EVO40 microscope (Zeiss, Jena, Germany). Prior to microscopic observations, the samples were coated with Au using a Balzers PV205P sputtering magnetron (Oerlikon Balzers Coating, Brügg bei Biel, Switzerland).

### 2.5. Tensile Test of Composites

Tensile tests were carried out using a ZwickRoell tensile machine, with a 10 kN load cell unit (Zwick GmbH & Co. KG, Ulm, Germany). Measurements were performed according to PN EN ISO 527–3 standard, at a crosshead speed of 200 mm/min under standard conditions of 23 ± 2 °C and 50 ± 5% relative humidity. Specimens with dimensions of 150 mm × 20 mm (length × width) were cut along the extrusion direction from the thin sheets for each formulation. At least 10 specimens from each sample series have been prepared.

### 2.6. Thermoforming of Polyethylene Composites

**Figure 1:**
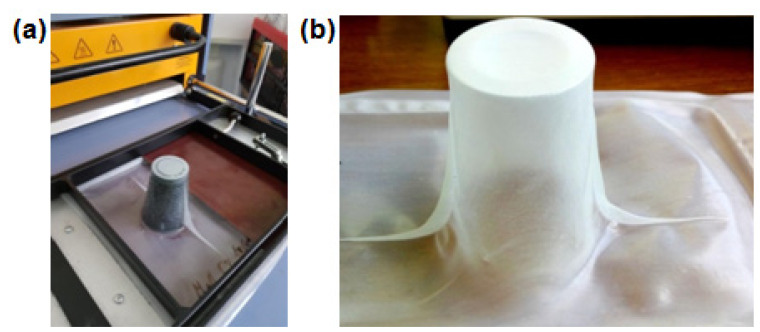
The thermoformed cup placed on a plug assist after the thermoforming operation (**a**) and webbing effect of the thermoformed cup (**b**).

The thermoforming process was carried out with a CR Clarke 725FLB vacuum thermoforming machine using the positive method. Due to the dimensional restrictions of film width, it was necessary to reduce the thermoforming field (see Figure 1a). The temperature of the heating plate was set at 190 °C, while heating time varied between 25–30 s. The thermoforming ability was checked using the positive method of thermoforming with a single-plug assist with its height of 84.5 mm and the outer diameter of 40 mm at the top and 60 mm at the bottom. In all thermoformed films, a webbing effect was observed (see Figure 1b), which could appear due to the longitudinal orientation of the films that were uniaxial-oriented during the casting and working of wind rolls. In order to determine the effect of film composition on the local thinning of thermoformed cup walls, measurements of the wall thickness were carried out using a Mitutoyo micrometer (Mitutoyo Polska Sp. zo.o., Wrocław, Poland).

### 2.7. Concact Angle Measurements

The wettability was determined using the Drop Shape Analysis System DSA100E (KRÜSS GmbH, Hamburg, Germany, accuracy ±0.01 mN/m). The calculation method (Young–Laplace) is based on the static contact angles (the sessile drop method is the standard method for an accurate wetting test). The drop is produced before the measurement and has a constant volume (2 µL) during the measurement. After successful fitting of the Young–Laplace equation, the contact angle was determined as the slope of the contour line at the 3-phase contact point.

The surface free energy (*σ_s_*) was determined with the Owens, Wendt, Rabel, and Kaelble method (OWRK), which is a standard method for calculating the surface free energy of a solid from the contact angle with several liquids – polar (water) and nonpolar (diiodomethane). The calculation of surface free energy (SEF) using the Young equations [42] (Equation (1)) and the Fowkes method [43] was carried out. The interfacial tension *σ_sl_* is calculated based on the two surface tensions *σ_s_* and *σ_l_* and the similar interactions between the phases. These interactions can be interpreted as the geometric mean of a disperse part *σ_D_* and a polar part *σ_P_* of the surface tension or surface free energy (Equation (2)).

(1) $$\sigma_{s}=\sigma_{sl}+\sigma_{l}\cdot \cos\theta$$

(2) $$\sigma_{s}=\sigma_{sl}+\sigma_{l}-2\left(\sqrt{\sigma_{s}^{D}\cdot \sigma_{l}^{D}}+\sqrt{\sigma_{s}^{P}\cdot \sigma_{l}^{P}}\right)$$

## 3. Results and Discussion

### 3.1. Microstructural Observations

In the literature, there is ample evidence that achieving good dispersion of lignin in polyolefins using simply melt mixing is difficult. The application of a long mixing time in a twin-screw extruder may cause lignin degradation, reflected as the presence of air bubbles and microvoids, as reported by Toriz et al. [44].

**Figure 2:**
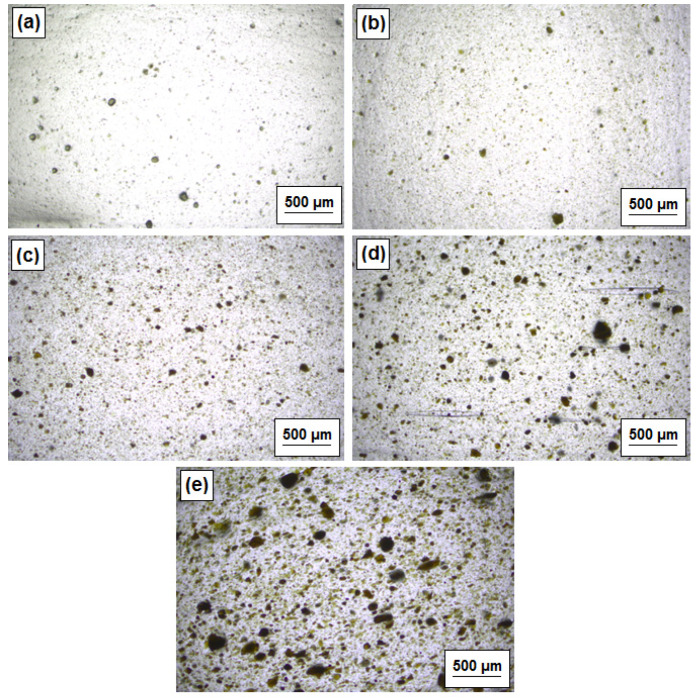
The distribution of MgO, lignin, and MgO-lignin hybrid particles in the LDPE matrix: LDPE/MgO (**a**), LDPE/MgO-L (5:1 wt./wt.) (**b**), LDPE/MgO-L (1:1 wt./wt.) (**c**), LDPE/MgO-L (1:5 wt./wt.) (**d**) and LDPE/lignin (**e**).

Images presented in Figure 2, obtained using the optical microscope, show the local morphology of LDPE composites with MgO, lignin, and hybridized particles that contain 5% by wt. of additives, where the lignin content in hybridized structures varied from 20% up to 80% by wt. of the total weight of filler. Typical observation of morphology resulted in an expected conclusion, that no or minor particles in the agglomerated form are visible after the application of compatibilizer and configuration of screws in the plasticizing unit of the Zamak extruder to achieve uniform filler distribution. As expected, along with an increase of lignin content in bi-component fillers, particles became bigger, and they are visible as a brown/dark stain because of the lignin concentration. Increased amounts of lignin in the polyethylene matrix resulted in stronger interparticle affinity and occurred in an aggregated microstructure.

**Figure 3:**
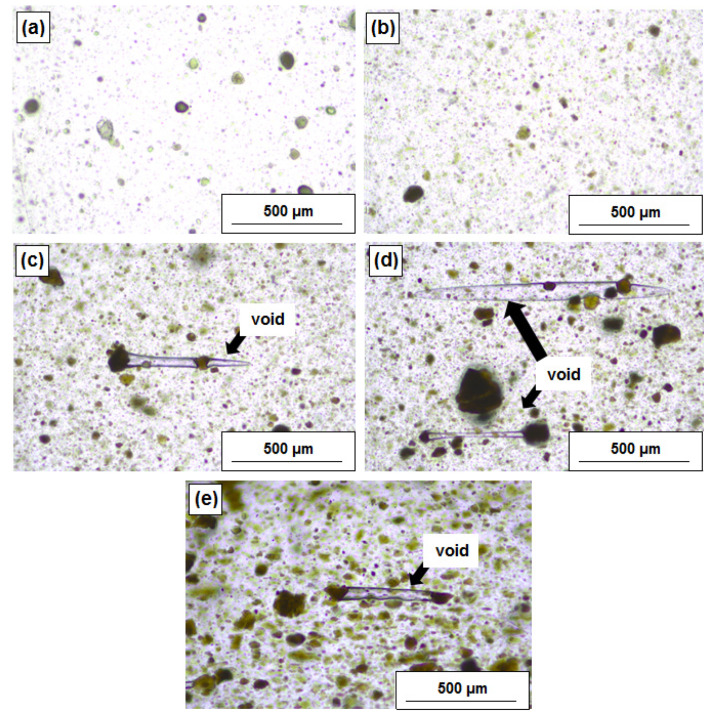
The distribution of MgO, lignin and MgO-lignin hybrid particles in the LDPE matrix observed under higher magnification: LDPE/MgO (**a**), LDPE/MgO-L (5:1 wt./wt.) (**b**), LDPE/MgO-L (1:1 wt./wt.) (**c**), LDPE/MgO-L (1:5 wt./wt.) (**d**) and LDPE/lignin (**e**).

To verify the effectiveness of mixing efficiency and local dispersion of dual fillers in the presence of a higher lignin amount, the same sheets were analysed with a twofold higher magnification (Figure 3). The images confirm that elongated voids appeared for the hybrid filler with a higher content of lignin and also for pristine lignin. Direct microscopic observations revealed that the mentioned pores are not created outside single lignin particles, but they are rather stretched into the form of elliptic voids between the particles. Therefore, the composites’ films do not exhibit regular porosities like foam or foamed microcellular thermoplastics. Those elliptic micro-voids resulted from the uniaxial stretching of viscous composites in the presence of hybridized fillers and lignin. This may suggest that the voids are created and localised around one single void and are continuously growing up to another neighbour particle. The mechanism of the void formation is less probable with filler degradation, but with lower adhesion on the polymer/filler interphase and local matrix deformation. To overcome such issues, a higher addition of the compatibilizer or surface modification of lignin is suggested based on a similar experience as it was examined in Reference [18].

Contrary to the above description, it can be proposed that the evolution of composite morphology from classical dispersion to that which includes numerous micro-voids as a function of lignin content may possess some positive aspects. In general, the formation of voids in the material leads to poor mechanical properties, and therefore we can expect a shorter lifetime for such a foil, used for example as a short-term packaging material in contact with food. This could bring new aspects and properties associated with accelerated decomposition caused by increased water intake by lignin.

This idea is only a hypothesis which should be checked, but the association between lignin content and void appearance is a noteworthy and promising aspect in such a case. On the other hand, it should be also pointed out that numerous voids may contribute to the breathable properties of the foil, which can be used for fruit/vegetable transportation and as an antifogging material.

**Figure 4:**
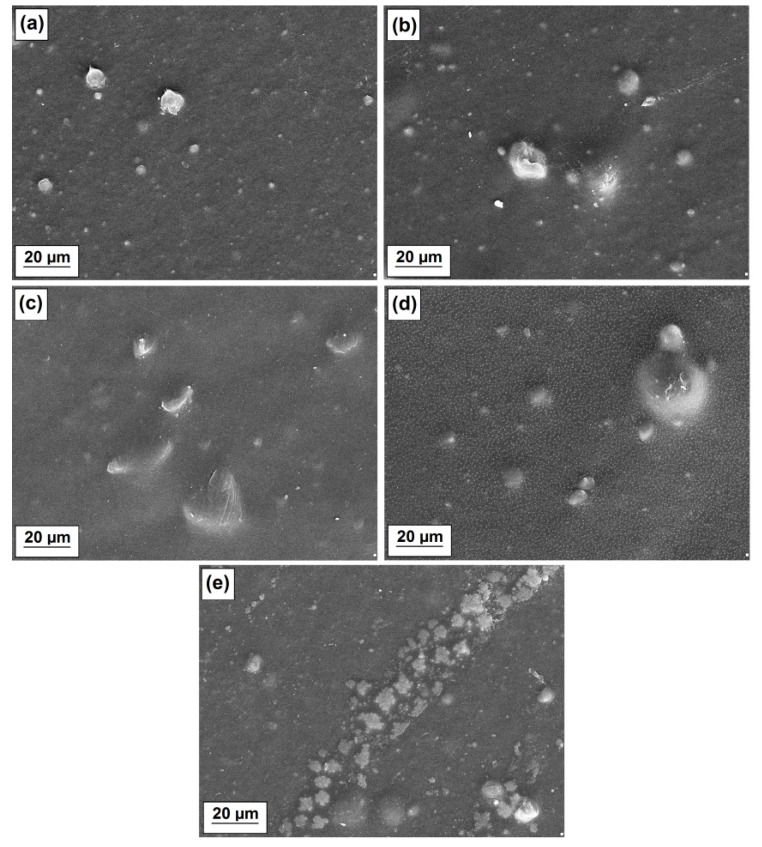
SEM images of obtained composites: LDPE/MgO (**a**), LDPE/MgO-L (5:1 wt./wt.) (**b**), LDPE/MgO-L (1:1 wt./wt.) (**c**), LDPE/MgO-L (1:5 wt./wt.) (**d**) and LDPE/lignin (**e**).

Further morphology investigations were carried out with scanning microscopy. Appropriate images are presented in Figure 4. As expected, the SEM technique provided mostly topography, in case of which the polymer matrix covers incorporated filler particles. Therefore, as can be clearly seen in Figure 4c,d, the inorganic phase is hidden and the assessment of morphology is difficult.

**Figure 5:**
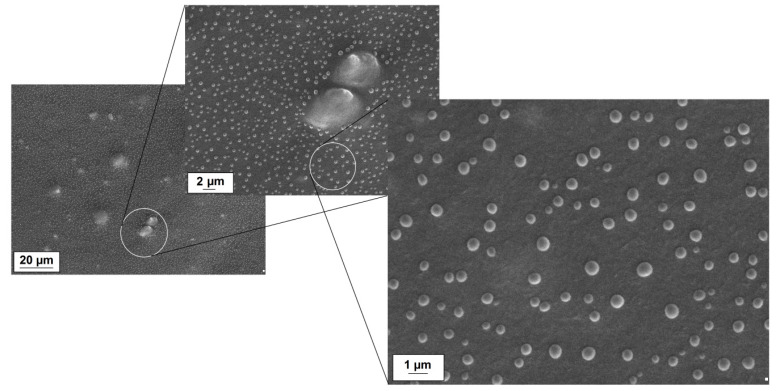
SEM images of the LDPE/MgO-L (1:5 wt./wt.) composite.

New information regarding microscopic morphology was obtained from a detailed observation of the LDPE/MgO-L (1:5 wt./wt.) composite surface. The application of a higher magnification allowed us to discover some unexpected micro droplets, visible in Figure 5. The droplets are spherical and, for most of the observable droplets, the diameter does not exceed 1 micrometer. Moreover, they are randomly distributed on the film surface, not aggregated. The micrograph suggests that droplets do not swallow the polymer surface. They might result from water condensation on the film surface.

### 3.2. Mechanical and Technological Properties of Thermoformed Polyethylene/Hybrid Composites

Some of the selected mechanical results are summarised in Table 1. As it was expected, the occurrence of micro-voids described in the previous subchapter, associated with increasing lignin content in composites, led to a deterioration of tensile strength. Relative to neat LDPE, the LDPE/MgO-L (1:5 wt./wt.) composite exhibited a 25% decrease in tensile strength, while in the case of the LDPE/MgO-L (5:1 wt./wt.) only a slight decrease was noted. This confrontation again shows the indirect relationship between the presence of lignin in compositions and the occurrence of voids, or in other words, tensile strength decrease. Interestingly, the increase of lignin content in hybrid fillers, as well as pristine lignin, resulted in a continuous increase of the Young’s modulus to almost twofold values compared to neat LDPE. Such a relationship between tensile strength and an increase of lignin concentration in LDPE as well as PP was also noticed by Iyer et al. [33]. The authors reported that, in order to achieve a major improvement of Young’s modulus in LDPE, they had to use 30% by wt. of rigid lignin particles in the composition.

**Figure 6:**
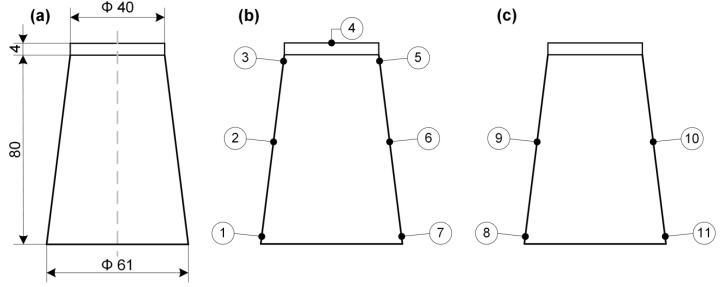
The main dimensions of plug-assist (**a**), the thermoformed cup and indication of points for thickness measurements in the extrusion direction (no. 1–7) (**b**), and indication of points for thickness measurements in the transferred direction to extrusion (no. 8–11) (**c**).

The ‘so-called’ thermoforming ability was checked using shaping in a positive-forming single plug assist. In this case, a male (positive) plug assist is pushed into the heated sheet before the vacuum is applied. This method allows for a better distribution of material, and deeper shapes can be formed, in case of which the depth-to-width ratio of more than 1:1 is possible, contrary to the negative-forming mode. Positive-forming plug-assisted forming applies a pre-stretch to the plastic sheet heated above the softening point and, therefore, improves the performance of the material and improves wall thickness distribution. In order to determine the effect of fillers on wall thinning of the product during thermoforming, eleven measuring points of the thickness of thermoformed films have been chosen. The wall thickness was measured by using a micrometer with an accuracy of 0.001 mm. Approximate locations for measuring points on the cross-section of the thermoformed shape are presented in Figure 6. The measuring points with numbers 1, 7, 8, and 11 were set at a 5 mm distance from the bottom, while measuring points 2, 6, 9, and 10 were established at 40 mm from the bottom. Finally, measuring points 3 and 5 were chosen at 70 mm from the basis of the shape. The results of the wall thickness of the thermoformed sheets with the positive forming method is presented in Table 1.

**Figure 7:**
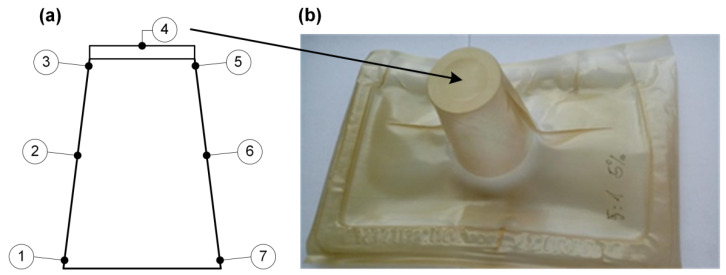
A drawing which illustrates point no. 4 set as a less-elongated (**a**) and thermoformed sheet with arrow indication of the top surface (**b**), where the wall thickness was close to the initial thickness of the film before the thermoforming process.

To compare the role of the fillers in the film thickness distribution in positive-formed shapes, the authors decided to show the relative wall distribution. In that case, the reference point and initial wall thickness were taken into account. After making all the necessery measurements for all tested shapes, including initial wall thickness, the thicknesses measured from point no. 4 corresponded closely to the value of the initial film thickness. Therefore point no. 4 was chosen as a reference point for the estimation of wall thinning. We think that during positive forming and film stretching, this is the place of the first contact with the male mold face. As a consequence, this area is not subjected to stretching. Moreover, we consider that after the first contact between the hot film and the male mold face, the coefficient of friction became high and the film cannot be moved and, therefore, massive wall thinning is avoided. That was the basis for the decision to choose point no. 4 as a reference point in the case of wall thickness comparison. The described procedure gives results of the wall thinning related to the initial thickness of the film. That assumption is correct if we agree that point no. 4, which represents the reference wall thickness, is only slightly elongated at the very beginning of the thermoforming process. In Figure 7, there is a picture of a real shape, formed in positive mode with an indication of the place where the reference point was established.

The results of the wall thicknesses measurements taken for shapes produced in the positive forming process and recalculated as a percentage contribution of initial film thickness, represented by point no. 4, are presented in Table 2.

The most visible percentage of wall thinning occurred for the LDPE/MgO-L (5:1 wt./wt.) composite film. The increase of the lignin amount in such a dual-component filler has a very positive influence on wall thickness distribution, which resulted in decreased thinning. Undoubtedly, the shape formed with LDPE/MgO-L (1:5 wt./wt.) composition is characterized by the best material arrangement and the highest mean wall thickness percentage. One of the possible explanations for such a coincidence is that the low molecular weight lignin polymer, which was spread out into the polyethylene matrix, may act as a softener that enables the achievement of more uniform sheet softening. On the other hand, thermal decomposition of lignin was restricted by the presence of magnesium oxide into the bi-component dual phase filler. Our previous studies confirmed the better thermal stability of the MgO-L systems against pristine lignin filler [26,27]. Moreover, a high number of aggregates visible in LDPE/lignin morphology, presented in Figure 2e, may effectively reduce the ability of such material to achieve uniform film thinning during stretching of the initial sheet.

### 3.3. Analysis of Wettability for Obtained LDPE Films

The wettability is an important property of synthesized polymers films because it allows us to determine the interactions between the solid and the liquids, and hence control the structure of the surface. The results of the contact angle measurements for analysed films are presented in Table 3.

The wettability analysis for the LDPE film indicated that the contact angle for the water was equal to approximately 102°, which is a similar value to that presented in the scientific literature [45]. To determine the influence of the addition of magnesium oxide and lignin, reference samples such as LDPE/MgO as well as LDPE/lignin were investigated. For the LDPE with magnesium oxide addition, a decrease of the water contact angle (92.08°) and hence surface free energy compared to LDPE was observed. For the second sample (LDPE/lignin) the contact angle for diiodomethane and water was equal to 44.91° and 74.06°; additionally, the SFE was 41.19°.

In the next step of wettability analysis, the hybrid materials, LDPE with the addition MgO-lignin, were analysed. In the case of LDPE/MgO-L (5:1 wt./wt.), the contact angle for water was similar to the reference sample LDPE/MgO; however, the contact angle for diiodomethane was decreased (48.8°), and hence the SEF has increased to a value 34.25 mN/m^2^. With the increase of the lignin content (sample LDPE/MgO-L (1:1 wt./wt.)), a similar value of the contact angle for water relative to the above-mentioned hybrid material was observed. Additionally, a change of the contact angle for diiodomethane (52.77°) and surface free energy (36.92 mN/m^2^) was noted. In the case of the LDPE/MgO-L (1:5 wt./wt.) hybrid material, the contact angle for water and diiodomethane was respectively equal to 53.91° and 32.23°. Furthermore, it should be noted that this material exhibited the highest SEF of all synthesized LDPE films, including pristine LDPE. This fact may be associated with the synergic effect between LDPE and the MgO-lignin hybrid material. Both for the pristine LDPE and LDPE with lignin, the surface free energy is high (37.19 and 41.19 mN/m^2^, respectively). Based on the literature reports, it is commonly known that some properties of synthesized materials can be improved in the chemical systems such as oxides or hybrid systems. Wysokowski et al. [46] indicated that the synthesis of chitin-POSS hybrid systems influenced the wettability. In this case, the improvement of hydrophobic properties was observed. Moreover, Sulym et al. [47] reported that by changing the mass ratio of multi-walled carbon nanotubes and poly(dimethylsiloxane), the wettability of the surface can be changed. Therefore, the synthesis of LDPE/MgO-lignin hybrid systems is indicated as the main factor influencing the wettability of the obtained LDPE films. Finally, according to scientific knowledge, the analysed LDPE films, in particular LDPE/MgO-L, have been recognized as materials with high wettability.

The research of Kasalkova et al. [48] and Parizek et al. [45] proved that in the case of polymer materials, their wettability is unsuitable for a wide range of applications such as tissue engineering, printing, and coating. Howarter and Youngblood [49] reported that the modification of various polymers surfaces with 3-aminopropyltriethoxysilane improves the hydrophobic properties of the analysed surface. However, the use of the above-mentioned organosilicon compound generates high costs, while in the green chemistry strategy, it is important to use low-cost materials. Therefore, there is a need to study new groups of fillers for polymers based on hybrid materials [27]. Moreover, Notley and Norgren [50] reported that the lignin had a high surface free energy at a low contact angle for water (about 50°), and hence it is an interesting material for modifying the polymer surface. Based on the mentioned literature review, the use of lignin has been justified; however, the authors also emphasize the use of magnesium oxide. The available scientific literature does not indicate any influence of MgO on wettability because the MgO in hybrid systems mainly improves the thermal stability [27] as well as increases of weld strength and the force needed for tear [26]. Although the effect of MgO on contact angle for water as well as surface free energy is not currently described in literature reports, it cannot be excluded. Based on the presented results, it should be noted that the sample LDPE/MgO-L (1:5 wt./wt.) is characterized by the best wettability, an additionally exhibits the highest surface free energy within all the analysed materials. The improvement of wettability can be explained by the synergic effect with lignin, and additionally, the MgO has numerous hydroxyl groups on the surface [51,52], which can allow for a decrease of the contact angle.

**Figure 8:**
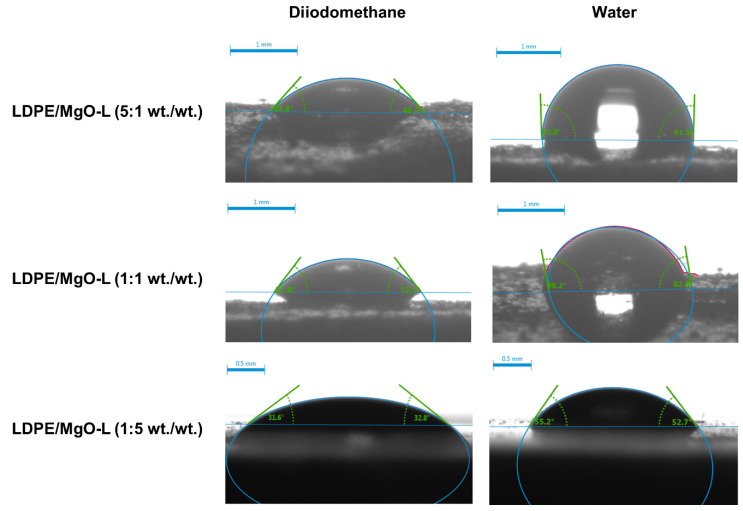
Diiodomethane and water contact angles for LDPE/MgO-L components.

The contact angle measurements for LDPE/MgO-L films are presented in Figure 8.

## 4. Conclusions

In this article, a new type of dual component MgO-lignin filler was used to modify the microstructure and thermoforming ability of low-density polyethylene. Three types of dual phase fillers with varying amounts of lignin phase, together with pristine lignin and MgO, were melt-mixed with LDPE, and thin sheet films were prepared as a semi-finished product.

Observation of surface morphology of semi-finished thin sheet films revealed a negative/positive microstructure with evidence of micro-voids, especially for the higher content of lignin. Micro-voids decrease the crack resistance of the semi-product.

The presence of micro-voids, especially in compositions with lignin as an additive, is the plausible reason which caused a substantial decrease in the tensile strength of the prepared films. However, rigid particles in any compositions resulted in a significant enhancement of Young’s modulus.

To compare the role of the fillers in the film thickness distribution in positive-formed shapes, the comparisons of relative wall distribution have been tested. The reference point and initial wall thickness were taken into account. The obtained results confirmed that the material composed of LDPE/MgO-L (5:1 wt./wt.) dual phase fillers exhibited the lowest thermoformability. In contrast, increasing the lignin amount in such a bi-component filler resulted in a very positive influence on wall thickness distribution (notable decrease of wall thinning). Therefore, the authors suggest research to develop other properties, especially for the most promising LDPE/MgO-L (1:5 wt./wt.) composite, considering its application in breathable and uniformly-shaped, partially bio-based packaging materials. Based on the results of the contact angle measurement, it was established that the analysed LDPE films are materials with high wettability. Furthermore, the effect of magnesium oxide content on the contact angle and surface free energy was observed, as higher SEF was noted in the case the LDPE/MgO-L (1:5 wt./wt.) sample than in reference samples (LDPE/MgO and LDPE/Lignin). Additionally, it was indicated that the improved wettability can be associated with the synergic effect of MgO and lignin in the hybrid system.

## Figures and Tables

**Table 1:** Mechanical results of tested films.

Sample Description	Tensile Strength (MPa)	Stand. Dev. (MPa)	Young’s Modulus (MPa)	Stand. Dev. (MPa)
LDPE	11.1	1.2	105	8.7
MgO	9.1	0.8	150	11.2
MgO-L 5:1 wt./wt.	10.5	1.1	176	15.4
MgO-L 1:1 wt./wt.	10.3	0.7	180	12.0
MgO-L 1:5 wt./wt.	8.4	0.9	188	14.1
Lignin	7.8	0.6	196	26.9

**Table 2:** Percentage contributions of relative wall thicknesses related to point no. 4.

Measurement Points	Samples Used for Thermoforming					
	LDPE	MgO	MgO-L 5:1 wt./wt.	MgO-L 1:1 wt./wt.	MgO-L 1:5 wt./wt.	Lignin
	Relative Wall Thickness (%)					
1	49.0	45.7	39.6	48.7	55.3	47.6
2	48.0	49.0	29.3	42.7	54.5	39.8
3	63.3	42.4	24.4	37.3	49.6	42.2
4 Reference point	100.0	100.0	100.0	100.0	100.0	100.0
5	50.7	49.5	46.3	52.0	65.7	66.5
6	64.0	47.6	43.3	64.0	61.9	56.8
7	60.1	37.1	40.2	81.3	59.3	47.1
8	46.3	53.8	50.6	49.3	65.2	43.2
9	48.0	48.1	41.5	43.3	64.8	41.3
10	30.0	37.1	40.9	27.3	35.8	26.2
11	33.5	29.5	31.1	30.7	35.0	38.8
Mean relative wall thickness	49.3	44.0	38.7	47.7	54.7	45.0

**Table 3:** Diiodomethane and water contact angles and the surface free energy of the adhesion of the samples.

Sample	Contact Angle (°)		SFE (mN/m^2^)
	Diiodomethane	Water	
LDPE	44.91 (±0.44)	102.24 (±0.36)	37.19 (±1.27)
LDPE/MgO	59.21 (±0.74)	92.08 (±0.18)	30.97 (±1.06)
LDPE/MgO-L (5:1 wt./wt.)	48.8 (±0.63)	90.59 (±0.47)	34.25 (±1.41)
LDPE/MgO-L (1:1 wt./wt.)	52.77 (±0.67)	89.64 (±0.56)	36.92 (±0.96)
LDPE/MgO-L (1:5 wt./wt.)	32.23 (±0.97)	53.91 (±0.44)	42.33 (±0.92)
LDPE/lignin	44.91 (±0.55)	74.06 (±0.26)	41.19 (±1.13)
